# Late Onset of the Fabella Syndrome after Total Knee Arthroplasty

**DOI:** 10.1155/2019/5219237

**Published:** 2019-11-04

**Authors:** Takeshi Kimura, Hidenori Tanikawa, Takayuki Hasegawa, Kentaro Takeda, Kengo Harato, Shu Kobayashi, Yasuo Niki, Kazunari Okuma

**Affiliations:** ^1^Department of Orthopedic Surgery, Saitama City Hospital, Saitama 2460, Mimuro Midori-ku, Saitama-shi, Saitama, Japan; ^2^Department of Orthopedic Surgery, Saiseikai Yokohamashi Tobu Hospital, 3-6-1, Shimosueyoshi, Tsurumi-ku, Yokohama-shi, Kanagawa, Japan; ^3^Department of Orthopedic Surgery, Keio University School of Medicine, 35 Shinanomachi, Shinjyuku-ku, Tokyo, Japan

## Abstract

Some differential diagnosis is thought due to knee pain after total knee arthroplasty (TKA) and fabella syndrome may cause post-TKA pain due to mechanical irritation. In this present case, a 64-year-old woman experienced lateral knee pain which was localized at the iliotibial ligament 8 years after the surgery. Fabella syndrome was diagnosed, and fabellectomy provided immediate resolution of the pain. The previous reports have revealed the symptoms occurred after 6 days to a year after total knee arthroplasty. This case widens the time span and the consideration of the fabella syndrome. The reason of this late onset symptom could be due to the enlargement of the fabella over time. We report that the differential diagnosis of fabella syndrome should be thought in symptoms of late onset knee pain after total knee arthroplasty.

## 1. Introduction

The fabella is a sesamoid bone that is located in the lateral head of the gastrocnemius muscle. Due to the impingement of such, fabella syndrome has been identified. It is uncommon, but relevant, which causes post-TKA pain due to mechanical irritation of the posterolateral tissues of the knee. We report the late onset of the fabella syndrome, which was difficult to diagnose.

## 2. Case Presentation

A 64-year-old woman underwent total knee arthroplasty for degenerative joint disease of the right knee in 2009. We used a posterior stabilizing type TKA (Balanced Knee System, Japan MDM Inc., Tokyo, Japan) with patellar resurfacing. We performed normal parapatellar approach and fixed the implants with bone cement. The standard rehabilitation included range of motion, muscle strengthening, and walking exercise under full weight bearing. Rehabilitation started from the first postoperative day.

Her condition was well through the first six months after the surgery. She had no pain, and the range of motion of the knee was full extension to 120-degree flexion. She was feeling slight and occasional pain in her knee while walking from 2010, approximately six months after the surgery. She had no inflammatory symptoms, and the radiographs of her right knee were normal without loosening or osteolysis at that time. Since the knee pain was tolerable and was not getting worse, we continued the outpatient follow-up once a year.

**Figure 1:**
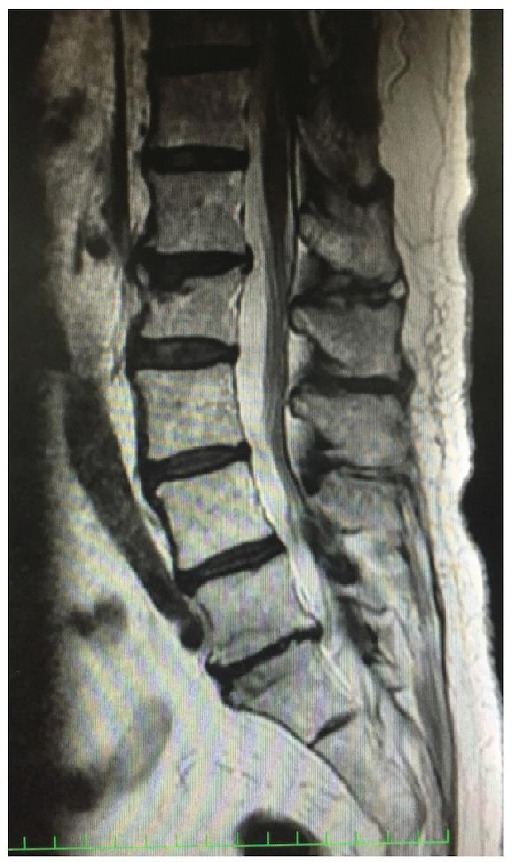
Lumbar disc herniation was thought as different diagnosis but was not apparent.

**Figure 2:**
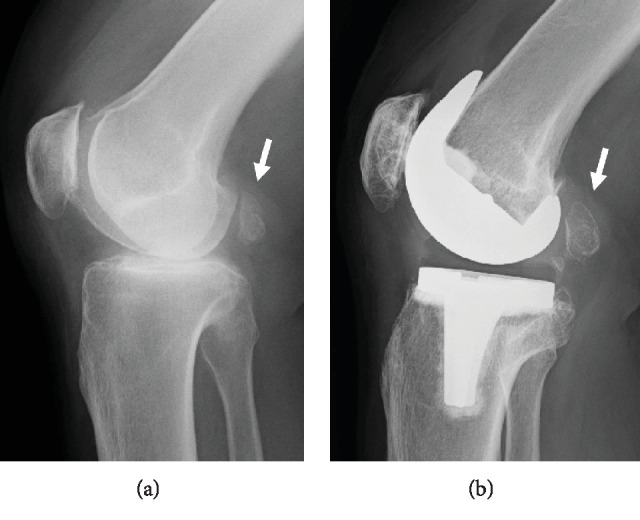
(a) Radiograph before TKA. (b) Enlargement of the fabella (arrow) is shown on radiograph 8 years after TKA.

**Figure 3:**
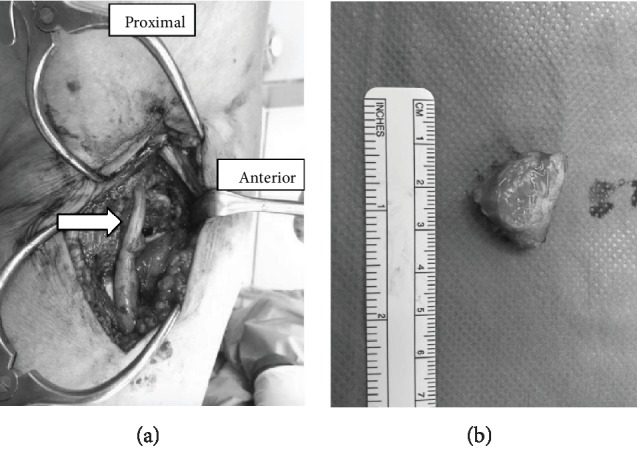
The peroneal nerve (arrow) was pushed by the fabella located just near the nerve. (b) Dissected fabella.

She had severe knee pain and came to our hospital again on March 2017, 8 years after the surgery. She was limping due to severe lateral knee pain localized at the iliotibial ligament. The pain increased when she walked and also increased when she extended her knee, as well as flexed her knee from full extension. The range of motion was from full extension to 130-degree flexion without any catching or clicking. No redness or swelling was observed. She did not have any numbness on her leg, but she felt pain on the lateral side of her shank when we hit an iliotibial ligament at the point around about 3 cm proximal from a fibula head. The strength of the tibialis anterior and the extensor hallucis longus was weak. The MRI of her lumbar was normal (Figure 1). Relatively, a large fabella with the size of 2 cm was found in the radiograph of her right knee (Figure 2). The radiographs were normal, and no loosening of the implants was observed. Finally, from these clinical and image features, we diagnosed her with a fabella syndrome and determined excision of a fabella (fabellectomy). The operation was performed using a posterolateral approach between the iliotibial tract and the biceps femoris. Macroscopically, the peroneal nerve was pushed by the fabella located just near the nerve (Figure 3). A posterior portion of the femoral implant was located under the fabella. The size of the fabella was 20 mm along the major axis, with osteophyte and deformation of the cartilage confirmed. Subjective symptoms resolved immediately after the surgery. At 1 month after the surgery, the range of motion was 0-130 degrees and the VAS score improved to 10 mm. She has no recurrence of snapping or pain on the posterolateral aspect of the knee.

## 3. Discussion

The fabella is a sesamoid bone that is located in the lateral head of the gastrocnemius muscle, and it has been identified on magnetic resonance imaging in 31% of Japanese people [1, 2]. The fabella usually ossifies at the age of 12-15 years, is present in 10-30% of individuals, and is bilateral in 80% of the cases [1]. Anatomically, the size of the ossified fabella can range from a pinpoint to 2.2 cm with a mean average size in adults of 1 cm [3]. The symptoms of fabella syndrome are posterolateral pain and clicking or a catching during the flexion of the knee. Jaffe et al. [4] were the first to report fabella impingement after total knee replacement. Larson and Becker [5] reported a patient with posterolateral knee pain, swelling, and catching of the fabella on the femoral component after a total knee replacement. In most cases, fabella syndrome is diagnosed radiographically with the symptoms, such as pain and clicking, and some can be diagnosed with the dynamic movement of the knee using ultrasonography [6]. The previous reports have revealed that the symptoms occurred after 6 days to a year after total knee arthroplasty [2].

In this case, the occasional lateral knee pain first occurred half a year after the surgery and improved naturally. Then, 8 years after the surgery, the symptom recurred, which in the terms of time, it is different and unique from the previous reports. The reason of these late-onset symptoms could be due to the enlargement of the fabella over time. The diameter of the fabella shown in radiography before surgery was 20 mm, whereas the diameter of the resected fabella was 25 mm. Also, the resected fabella contained many osteophytes around it. Previous reports said that the anterior surface is covered with hyaline cartilage [7].

Apparently, lumbar disc herniation was suspected. And also, MMT of the lower leg was dominantly depressed, but the MRI revealed no sign of herniation. The tinel-like syndrome she felt when we hit the iliotibial ligament suspected us into the fabella syndrome. Nerve conduction test may have given us a more specific detail of the entrapment of the nerve.

Also, in the radiographs, the distance between the component and the fabella is near, and the enlargement of the fabella through time might be a clue to suspect this disorder (Figure 2). Fabellectomy is useful for the treatment.

In conclusion, we report that the differential diagnosis of fabella syndrome should be thought in symptoms of late-onset knee pain after total knee arthroplasty.
